# Sensory properties of Australian bunya nuts

**DOI:** 10.1111/1750-3841.16184

**Published:** 2022-05-20

**Authors:** Jaqueline Moura Nadolny, Odette Best, Emma Hassall, Heather M. Shewan, Sandra M. Olarte Mantilla, Jason R. Stokes, Heather E. Smyth

**Affiliations:** ^1^ School of Chemical Engineering The University of Queensland Brisbane Queensland Australia; ^2^ School of Nursing and Midwifery University of Southern Queensland Ipswich Queensland Australia; ^3^ Centre for Nutrition and Food Sciences, Queensland Alliance for Agriculture and Food Innovation The University of Queensland Brisbane Queensland Australia

**Keywords:** Araucaria, Australian native foods, bunya nuts, descriptive analysis, sensory evaluation

## Abstract

**Abstract:**

Bunya nuts are the seeds of *Araucaria bidwillii*, a conifer native to South‐East Queensland, Australia. They are one of the 19 species of Araucaria family found around the world, with the nuts from South America being the most commonly consumed. They are traditionally eaten boiled or roasted. This study aims to profile the sensory properties of bunya nuts with chestnut as a comparator. Since chestnuts do not come from a conifer tree, it is expected that there will be differences. Different methods of preparation are also expected to change the sensory attributes. Representative samples were collected from a variety of locations in South‐East Queensland, prepared and presented to a panel of 14 experienced tasters applying conventional sensory descriptive profiling.

During training, the panel developed a lexicon of 23 sensory attributes together with definitions and reference. Profiles of the boiled and roasted bunya nuts revealed higher scores for *hardness* on the first bite than chestnuts and, when chewed, became more *crumbly*, *dry*, and *grainy*. They had a *savory* aroma and flavor, and roasted samples exhibited a *roasted* aroma. Bunya nut samples were less sweet than chestnut samples. Differences in the sensory properties due to method of preparation were also observed. Boiled bunya nuts were softer and moister, with lower scores for *crumbly* and *grainy*. This research is foundational in providing technical information on the sensory profile of this important Indigenous Australian nut and provides a strong basis to support novel food sector opportunities for the bunya nut as a reemerging food source not only in Australia, but also South America.

**Practical Application:**

There is an increase demand for local, sustainable, and natural foods. Bunya nuts are native to Australia and are part of the Araucaria family, which includes 19 species that can be found around the world. To the best of our knowledge there is no study characterizing Araucaria nuts in terms of sensory attributes. This study builds a lexicon for bunya nuts and compares to chestnuts. It also shows how different preparation methods affect its sensory attributes, as well as possible future uses in product development. The outcomes might provide information to support studies on Araucaria nuts in other countries.

## 1 INTRODUCTION

Araucaria trees are a type of conifer found in the Southern hemisphere that some suggest have been around for more than 200 million years (Zonneveld, 2012). Bunya nut is the seed of *Araucaria bidwillii* and it is native to South‐East Queensland, Australia. The tree produces cones that fall once a year—during summer—and break open, releasing the nuts (Burrows et al., 1992). Traditionally, the nuts were consumed roasted on fire or fermented on the ground for several months (Vesoul & Cock, 2012). Recently, they have also been consumed boiled in water (Huth, 2002).

Araucaria species from Brazil (*Araucaria angustifolia)* and Chile (*Araucaria araucana)* also produce edible nut‐like seeds (Da Silva et al., 2014; Dos Reis et al., 2014). The bunya nut differs from the other species’ nuts in appearance, such as color and size, and studies on the nutritional properties of the nuts also show differences in composition, such as fat content (2.50 g/100 g of dried nut for *A. angustifolia* and 1.11 g/100 g of dried nut for *A. araucana*) and starch content (71.84 and 63.67 g/100 g of dried nut for *A. angustifolia* and *A. araucana*, respectively) (Cordenunsi et al., 2004; Henriquez et al., 2008). Bunya nuts traditionally were widely consumed and highly valued by First Nations Australians within Queensland and remain highly revered and consumed by First Nation people today. Among settler populations the timber was valued as a hard wood which led to great swathes of deforestation which has impacted First Nations consumption of this traditional food.

Currently, the nuts remain undervalued by the mainstream food‐value chain and sometimes the cones, after falling, are discarded as waste. Research on the sensory characteristics of bunya nuts, and the sensory impact of different preparation methods, will increase interest in these traditionally valued nuts. Such knowledge may also support Indigenous enterprise development and provide further scientific evidence of the value of bunya nuts as a delicious and nutritious food source.

There is limited research detailing the nutritional aspects of Araucaria nuts as well as processing and use of by‐products (Angélica Koehnlein et al., 2012; Conforti & Lupano, 2011; Daudt et al., 2014; Santi‐Gadelha et al., 2006; Santos et al., 2018; Spada et al., 2013). To our knowledge, there is no published research on the sensory profiles of the nut of any of the Araucaria species, although there is limited research on Araucaria nut products and ingredients (Ikeda et al., 2018). One such sensory study demonstrated consumer acceptance for the color, flavor, and texture of *A. angustifolia* extruded flour and revealed the flour product developed a natural flavor during processing (Boff Zortéa‐Guidolin et al., 2017). Another study evaluated the consumer acceptability of bread made with *A. angustifolia* flour, showing that the flour could be an acceptable alternative in the gluten‐free flour market (Pinto Polet et al., 2019).

Bunya nuts resemble chestnuts, not only by the fact that they are starchy and have lower amounts of fat (Vesoul & Cock, 2012) when compared to other types of nuts (Borges et al., 2008), but especially due to the way they are prepared and eaten, by boiling or roasting the nut with the husk, followed by peeling. However, since chestnuts come from a different family of trees, differences are expected. Different cultivars of North American chestnuts have been characterized in terms of sensory properties and key descriptive aroma and flavor attributes included *nutty*, *earthy*, *maple*, *sweet*, among others. Textural attributes for chestnuts included *firmness* and *dissolvability*. Importantly, significant differences in sensory properties were found in chestnuts grown in different regions, ostensibly due of the different climatic conditions of the regions where the chestnuts were grown: North and South of the United States (Warmund et al., 2011).

This study primarily aims to establish a lexicon for describing the sensory properties (textural, aroma, flavor, and aftertaste) of bunya nuts (from South‐East Queensland, Australia) in comparison to chestnuts and, second, to explore the sensory impact of two different processing and preparation methods to produce roasted and boiled nuts. Changes in sensory attributes are expected due to different moisture contents of boiled and roasted nuts and also due to Maillard reactions when the nuts are roasted. Conventional descriptive analysis was applied using an experienced trained sensory panel to provide a comprehensive and informative qualitative and quantitative evaluation of the bunya nuts (Lawless & Heymann, 2010). The outcomes of this study will be foundational in providing technical information on the sensory profile of this important Indigenous Australian nut and will provide a strong basis to support new food product opportunities and initiatives for the bunya nut.

## 2 MATERIALS AND METHODS

### 2.1 Samples

**FIGURE 1:**
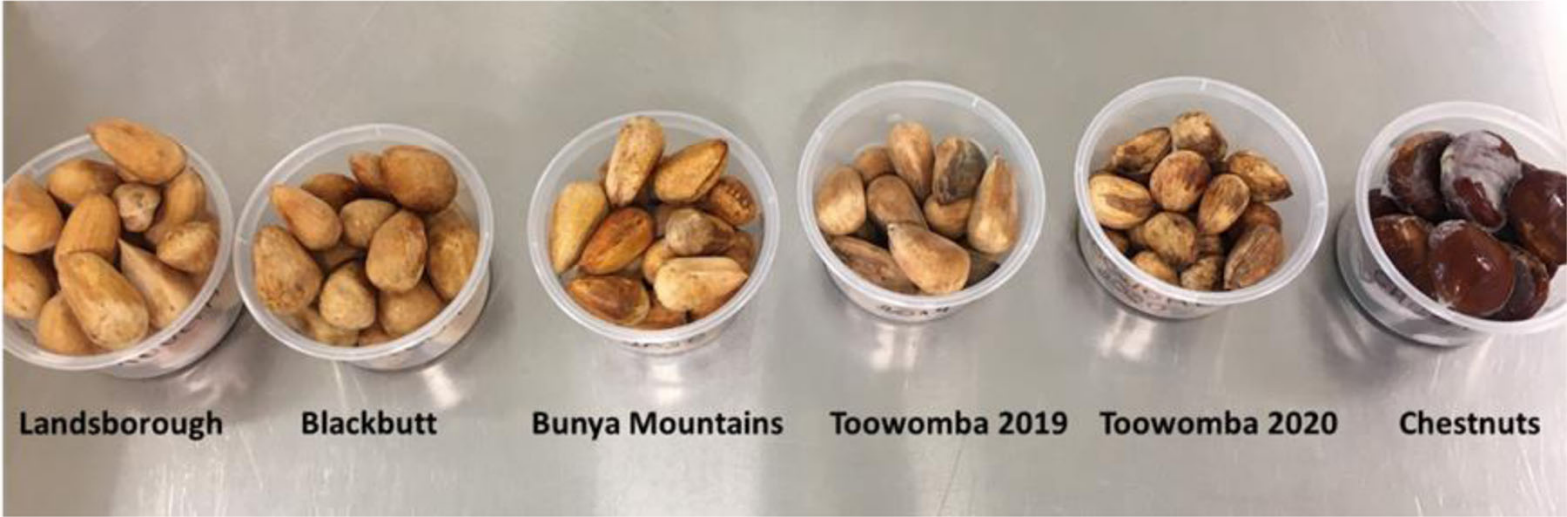
Photographs before cooking and husk removal of the five bunya nut samples and the chestnut sample evaluated in the study

A total of 17 kg of representative commercial samples of raw bunya nuts, equivalent to approximately 980 nuts, were collected or purchased from four different regions of South‐East Queensland, Australia (Bunya Mountains, Landsborough, Blackbutt, and Toowomba) and during two different seasons (Toowomba region—2019 and 2020, all other regions—2020 season only). The nuts were washed, dried at room temperature for 24 h, hand selected to remove damaged nuts, sealed in polyethylene bags under vacuum (each bag containing approximately 400 g), and stored (−18°C) until required. A total of 3 kg of chestnuts, equivalent to approximately 200 units, were purchased from Rocklea Markets, Queensland, Australia, and were similarly sealed and stored (−18°C), with each bag also containing approximately 400 g. The bunya nut samples were collected from different regions and seasons to ensure that the natural variation could be captured in this sensory study. A list of samples collected for evaluation is given in Table 1 with accompanying pictures of the raw nuts in Figure 1 (cooked nuts not shown).

**TABLE 1:** List of samples used in the sensory study

Type	Year	Region	Method of preparation	Sample
Bunya nuts	2020	Bunya Mountains	Roasted	1
			Boiled	2
		Landsborough	Roasted	3
			Boiled	4
		Blackbutt	Roasted	5
			Boiled	6
		Toowomba	Roasted	7
			Boiled	8
	2019	Toowomba	Roasted	9
			Boiled	10
Chestnuts	2020	Brisbane	Roasted	11
			Boiled	12

### 2.2 Sample preparation and presentation

The nuts were prepared following typical preparation methods of Araucaria nuts (Gama et al., 2010) for boiling and roasting as follows.

#### 2.2.1 Boiling protocol

The bunya nuts and chestnuts with the husk were cut in half across the length using a bunya cutter (a purpose‐designed tool purchased from Bevin Mutch in Maleny, QLD) and the nuts (∼200 g) were brought to boil in 1.8 L of water in a 2.5 L stainless steel pot on a gas cook‐top (Fisher & Paykel). Once boiling, the heat was lowered to obtain a gentle simmer with lid on (slightly ajar) (45 min). Subsequently, the nuts were cooled in cold running tap water (1 min), peeled, directly distributed into coded (three‐digit blinding code) polypropylene sample cups (45 ml) with lids, and stored (4°C) until required for sensory evaluation.

#### 2.2.2 Roasting protocol

The bunya nuts were cut in half and the chestnuts were cut in a shallow cross format through the flat side of the husk. This method prevented the nuts from overheating and bursting while cooking. After preheating the UNOX convection oven model XF135 (180°C) for 10 min, the nuts were placed on an aluminum tray lined with baking paper and subsequently heated in the oven (15 min) under humidity (40%). The trays were removed and the nuts were cooled to room temperature, peeled, directly distributed into coded (3‐digit blinding code) polypropylene sample cups (45 ml) with lids, and stored (4°C) until required for sensory evaluation.

#### 2.2.3 Sample presentation

All roasted or boiled nuts were evaluated by the trained panel within 15 h of cooking. Approximately 5 g of cooked nut was presented per sample representing two cut halves for bunya and two nut halves for chestnuts. Samples were removed from cold storage (4°C) 45 min prior to session and served at room temperature. Samples were presented to panelists for assessment on white trays. During formal evaluation, the 12 samples were assessed in quadruplicate and no more than 16 samples were assessed within a 2 h period.

### 2.3 Physicochemical analysis of the samples

Physicochemical analysis of the boiled and roasted bunya nut kernels was performed at an accredited laboratory (National Association of Testing Authorities [NATA], Symbio Alliance, Eight Mile Plains, Queensland, Australia). The following analyses were performed according to Association of official analytical collaboration (AOAC) methods: protein (AOAC 990.03 and 992.15), crude fat and oil (AOAC 960.39), moisture (AOAC 925.10 & 950.46), ash (AOAC 923.03 and 920.153), sugar profile by high performance liquid chromatography, dietary fiber (AOAC 985.29 & 991.42), and starch (AOAC 996.11 & AACC 76.13). The results are presented as the average of duplicates. The standard deviation was not provided by Symbio Alliance, although this value is always within 10% of variation between repeats as a requirement in this laboratory.

### 2.4 Sensory panel and evaluation method

Prior to formal panel evaluation, a bench‐top tasting involving five experienced panelists was conducted in order to determine the suitability of samples for presentation to the trained panel, to identify relevant attributes, and to develop sample presentation protocols including palate cleansers.

#### 2.4.1 Trained sensory panel and facility

The sensory panel was selected externally based on availability of individuals from a pool of panelists who had been previously tested for sensory acuity (Meilgaard et al., 2006) and were experienced in sensory descriptive studies. Three male and eleven female panelists, aged 19−66 years old (mean age of 41) participated in all training sessions (10 h, five sessions) and formal evaluation sessions (6 h, three sessions). Sessions were held in the sensory laboratory of the Health and Food Sciences Precinct, Coopers Plains, Queensland, which is equipped with 12 isolated sensory booths which are temperature controlled (22°C) and under daylight equivalent lighting.

#### 2.4.2 Sensory evaluation method

Conventional sensory descriptive analysis was the profiling method employed for evaluation of the 12 nut samples (Lawless & Heymann, 2010). During the training phase sensory vocabulary, definitions, reference standards, attributes scales, anchors, and method of assessment were generated by consensus, discussed, and optimized. The sensory properties evaluated were aroma, flavor, texture, and aftertaste. A total of 23 attributes were generated (seven aroma, five texture, six flavor, five after taste and mouthfeel after swallowing) and attribute definitions, together with reference standards are provided in Table 2. Attributes *other aroma* and *other flavor* were included in addition to the 23 attributes rated in this study for use if panelists experienced an unusual odor or flavor in any of the samples presented. This was deemed necessary given the natural variation among the nut samples. At the end of training, a practice session was held to collect preliminary data on panel performance and to ensure that the protocol and method presented were appropriate and clear.

**TABLE 2:** Sensory attributes and reference standards agreed by the trained panel for the bunya nut and chestnut samples

Attributes	Definition	Reference standard
**Aroma**		
*Aroma intensity*	The overall aroma intensity of the sample	Nil
*Sweet note*	The sweet aroma associated with sweet potato, pumpkin, caramel, or maple syrup.	Four drops of maple syrup (Green's)
*Roasted*	A roasted aroma, like toasted nuts, roasted chicken‐skin, toast, or popcorn.	One salted popcorn (Cobs)
*Savory*	A savory aroma like cooked potato, poached chicken, and broth.	One teaspoon of chicken salt (Nice n'tasty) mixed with 1 cm^3^ of cooked potato
*Herbal*	An herbaceous aroma associated with fresh parsley, a hint of eucalyptus.	Parsley mixed with one drop of an eucalyptus oil solution (five drops of oil in 700 ml of water)
*Earthy*	The earthy aroma associated with root vegetables or raw mushroom.	One tablespoon of soil with a slice of raw mushroom and 0.5cm^3^ of cooked carrot
*Chemical*	A chemical aroma, plastic‐like.	Natural candle mixed with polystyrene
*other aroma*	Any other aromas detected. Panelist to describe.	–
**Texture**		
*Hardness*	The force required to bite through the largest part of the sample using the front teeth (first bite). Low being soft, to high being very hard.	Brazil nuts
*Dry*	The perceived dryness of the sample. Low being moist, to high being very dry.	–
*Crumbly*	The degree that the sample crumbles on the first few bites.	macadamia nuts
*Floury*	The sensation of fine floury particles left in the mouth at the end of mastication.	Half teaspoons of tapioca starch in 8 ml of water
*Grainy*	The sensation of grainy bits left in mouth after chewing.	Macadamia nuts blended for 15 s
**Flavor**		
*flavor intensity*	The overall flavor intensity of the sample.	Nil
*Sweetness*	The sweet taste associated with sweet potato, pumpkin, caramel, and maple syrup.	One teaspoon of honey
*Savory*	A savory rich taste like cooked potato, poached chicken, and broth.	As in aroma
*Herbal*	An herbaceous flavor associated with fresh parsley, a hint of eucalyptus.	As in aroma
*Earthy*	The earthy flavor associated with root vegetables or raw mushroom.	As in aroma
*Chemical*	A chemical flavor, plastic‐like.	As in aroma
*other flavor*	Any other flavors detected. Panelist to describe.	–
**After taste and mouthfeel after swallowing**		
*hard to clear*	How difficult it is to clear the sample from the mouth and teeth. None being easy to clear, to high being difficult to clear.	–
*sweet linger*	A sweet lingering flavor after swallowing.	–
*Numbing*	A numbing sensation, mouth‐tingle, almost metallic.	–
*Drying*	A drying astringent sensation on the oral surfaces after swallowing.	Skim milk (Coles)
*Earthy*	An earthy flavor sensation after swallowing associated with root vegetables and raw mushroom.	As in aroma and flavor

For formal evaluation, the method of assessment was as follows: lift lid and assess the aroma, take a bite from the largest and through the widest part of the sample with your front teeth and chew to assess texture, take the second half into the mouth and assess flavor, assess aftertaste and mouthfeel after swallowing sample, rinse palate with water, rest for at least 45 s before assessing next sample, repeat as required. Filtered tap water was served as palate cleanser.

During formal evaluation, samples were presented according to a randomized complete block design (RCBD) with 16 samples presented per session (2 h) on separate days such that data for one and a third replicates were collected per session over 3 days. During sessions, a minimum break of 45 s was maintained between each sample. Attribute scores were collected using Redjade Sensory Software (Curion, Redwood City, CA, USA). Scales (0−100) were used with anchors “none” (0) to “high” (100) for all attributes except for *hardness*, (0: soft, 100: very hard), *dry* (0: moist, 100: very dry), and *hard to clear* (0: easy to clear, 100: difficult to clear) (Table 2).

### 2.5 Data analysis

Data were exported from Redjade to Microsoft Excel and XLSTAT (2019.4.2, Addinsoft 1995–2020, Paris, France) was used for product characterization, multivariate data analysis, and to analyze panelist performance. For all sensory attribute scores, minimum, maximum, mean, standard deviation, and coefficient of variation were calculated. Factors and interaction effects were analyzed using mixed model analysis of variance applied on the raw data set for each attribute to determine significant differences. Analysis of variance (ANOVA) was also performed on the sensory scores provided by each panelist to test discrimination power and repeatability of the panelists. A Pearson correlation coefficient was performed for each of the sample sets. Finally, a principal component analysis (PCA) was conducted on the mean scores for all samples to visually observe sample grouping, differentiation and to explore the samples sensory profiles.

## 3 RESULTS AND DISCUSSION

The results obtained include the characterization of bunya nuts as well as an analysis of panel performance and robustness of data. Differences in sensory characteristics found between bunya nuts and chestnuts and between methods of preparation are also discussed.

### 3.1 Physicochemical characteristics of boiled and roasted bunya nuts

The composition of boiled and roasted bunya nuts and the typical composition of chestnuts (Gonçalves et al., 2010; Li et al., 2016) are presented in Table 3.

**TABLE 3:** Proximate composition (g/100 g of sample of dried sample) of boiled and roasted bunya nuts and chestnuts samples

		Dry Matter^1^	Moisture^1^	Protein	Fat	Ash	Dietary Fiber	Starch	Glucose	Sucrose	Fructose
**Bunya nuts**	Boiled	44.7	55.3	4.2	1.3	2.3	7.6	65.8	0.4	4.2	0.6
	Roasted	60.4	39.6	4.4	2.7	2.4	7.5	65.4	0.4	5.2	0.5
**Chestnuts** ^2^, ^3^	Boiled	42.1	57.9	6.3	3.3	1.8	15.4	56.7	0.2	5.9	0.1
	Roasted	54.2	45.8	6.7	3.1	2.1	20.1	61.2	0.2	9.2	0.2

^1^ g/100 g sample;

^2^ Goncalves et at., 2010;

^3^ Li et al., 2016.

The values found for the composition of bunya nuts are in accordance with previously reported values for the other two Araucaria species from Brazil and Chile (Cordenunsi et al., 2004; Henriquez et al., 2008). The nuts differ from other types of nuts such as almonds, macadamias, and pine nuts as they are high in starch and moisture, rather than fat. The differences in composition between boiled and roasted bunya nuts are mainly related to moisture, by reason of absorbing and losing water during boiling and roasting, respectively.

The main difference found between bunya nuts and chestnuts is the amount of soluble sugars they contain. Chestnuts have a total of 6.2 g/100 g of dried sample in boiled nuts and 9.61 g/100 g for roasted nuts, while bunya nuts have 5.2 g/100 g and 6.1 g/100 g, respectively. The amount of each type of sugar is slightly different, with bunya nuts presenting more fructose and glucose but less sucrose. Also, chestnuts are slightly higher in moisture, protein, and fat, and have around twice as much dietary fiber. Bunya nuts are higher in starch and ash. The small differences on the sugar profile of boiled and roasted samples may be related to sucrose decomposition upon heating (Li et al., 2016), especially considering that the nuts were boiled three times (45 min) than roasted (15 min).

### 3.2 Panel performance and robustness of the sensory data

Prior to analyzing and making interpretations about the samples, panelist performance was examined in terms of discrimination ability among samples and repeatability across replicates to ensure the robustness of the sensory data. These results can be found in the Supporting Information (Table S1). Discrimination power is the ability of the panelist to differentiate among samples (Meilgaard et al., 2006). Repeatability determines how the panelist agrees in the assessment of the same sample throughout the formal sessions (Meilgaard et al., 2006). Trained panelists are an essential part of the statistical power of the study, since they practice to use attributes and scale similarly to others, avoiding high variance among replicates for example (Lawless & Heymann, 2010). Overall, the panel had good discrimination power and repeatability (Table S1), which means the evaluation of results is reliable.

A summary of the minimum, maximum, mean, standard deviation (SD), coefficient of variation (CV %), and standard error of the mean (SEM) for each of the sensory attributes were calculated to determine panel performance in terms of how the samples were distributed and how the scales were used. This information can also be found in the Supporting Information (Table S2). The 23 attributes used covered the sample differences well and could be used to differentiate them. Overall, the scale was well used for most attributes and variability was observed among samples (Table S2).

Table 4 shows the *F* ratios and significance for effects of the 12 samples. Aroma attribute *sweet note* and flavor attribute *savory* had the lowest contribution to the discrimination. This could be an indication that the panelists did not perceive much difference among samples for these attributes.

**TABLE 4:** *F*‐ratios and significance for effects of samples, panelist and replicate (12 samples × 4 replicates × 14 panelists)

Sensory attribute	Sample	Panelist	Replicate
*Aroma intensity*	11.4***	6.1***	0.47 ns
*Sweet note*	1.1 ns	3.7***	0.71 ns
*Roasted*	4.7***	5.4***	1.06 ns
*Savory aroma*	2.4**	10.1***	0.74 ns
*Herbal aroma*	3.5***	10.4***	1.29 ns
*Earthy aroma*	7.2***	2.6**	1.43 ns
*Chemical aroma*	2.4**	6.9***	0.35 ns
*other aroma*	6.8***	3.7***	0.74 ns
*Hardness*	176.8***	6.6***	5.21***
*Dry*	66.1***	4.3***	1.16 ns
*Crumbly*	4.6***	3.8***	1.24 ns
*Floury*	5.4***	5.3***	1.45 ns
*Grainy*	40.3***	9.1***	1.26 ns
*Flavor intensity*	25.2***	5.6***	0.28 ns
*Sweetness*	27.9***	5.8***	2.64*
*Savory*	1.1 ns	7.8***	0.36 ns
*Herbal*	4.0***	16.0***	1.56 ns
*Earthy*	8.3***	6.7***	0.75 ns
*Chemical flavor*	3.2***	12.5***	0.40 ns
*other flavor*	6.4***	4.8***	1.18 ns
*Hard to clear*	19.0***	7.4***	0.80 ns
*Sweet linger*	23.2***	4.0***	2.48*
*Numbing*	2.9**	19.6***	1.45 ns
*Drying*	8.5***	11.4***	1.51 ns
*Earthy aftertaste*	9.2***	11.0***	0.60 ns

Significant *F*‐ratios are indicated by

* (*p* < 0.1),

** (*p* < 0.01),

*** (*p* < 0.001) and ns: not significant (*p* > 0.1).

Most of the samples had high scores for aroma intensity. In terms of texture, differences in *hardness*, *dry*, and *grainy* were clear as the scale was broadly used. Except for *savory*, the flavor attributes were also well differentiated. Differences in aftertaste and mouthfeel were more meaningful for *sweet linger*, where the scale was used very broadly. However, all of them showed a degree of variation.

There were differences among panelists for each attribute (Table 4), indicating that panelists were using the attributes differently, which is typical for descriptive sensory data, since it may be dependent on mood, motivation, physiological aspects, hunger, and even familiarity with the product (Lawless & Heymann, 2010).

The rating for each replicate was not statistically significant for most of the attributes (Table 4), except for *hardness*, *sweetness*, and *sweet linger*, which means the replicates were similar among themselves and the method of preparation was well followed. *Hardness* depends largely on the roasting method. If the sample is slightly smaller, it will dry faster and probably be harder to bite. *Sweetness* and *sweet linger* are related with each other and also varied among replicates. The sensory panel performance and robustness of the sensory data were considered satisfactory and suitable for further data evaluation. Comparison among samples for each of these attributes is showed in the next section of the paper.

### 3.3 Overview of the sensory profile

**FIGURE 2:**
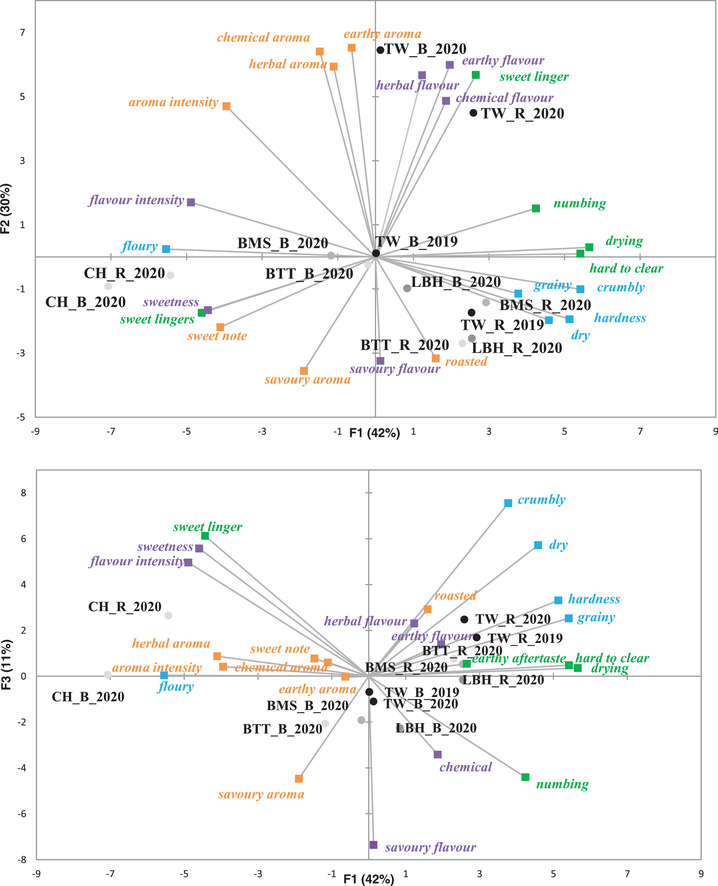
PCA bi‐plots of the sensory properties of 12 samples (*n* = 4 replicates × 14 panelists)

The PCA bi‐plot with the different attributes and the 12 samples is shown in Figure 2. The first two principal components *X* and *Y* axis explain 72% of the variation in the data. The first principal component (*X* axis) was driven by texture and aftertaste with *numbing*, *drying*, *hard to clear*, *crumbly*, and *hardness* having high positive loadings on PC1 and *floury* negative loadings. *Flavor intensity*, *sweetness*, and *sweet linger* also had high negative loadings on PC1. Chemical, earthy, and herbal aromas and flavors, and *earthy aftertaste* were all combined with high positive loadings on PC2, whereas savory flavor and aroma, and roasted aroma had negative high loadings on PC2.

The PCA plot suggests that four groups could be formed. Chestnuts were separated from bunya nut samples across PC1, correlating strongly to *sweetness*, *sweet linger*, *sweet note*, *flavor intensity*, and *floury*. Bunya nut samples collected in 2020 in Toowomba formed a second group across PC2 with high positive loadings for PC2 and correlation to herbal, chemical, and earthy aromas and flavors. In general bunya nut samples correlated to *savory* and *roasted*. Roasted bunya nut samples had slightly positive loadings for PC1 and high correlations to *dry*, *grainy*, *crumbly*, and *hard*, while boiled bunya nuts had almost zero loadings.

The third principal component (Figure 2) explained an additional 11% of the variance and confirmed the differences between chestnuts and bunya nuts as well as between roasted and boiled samples identified in PC1 and PC2. However, bunya nut samples collected from Toowomba in 2020 are not as separated from the other bunya nut samples as in PC3. Toowomba samples from 2020 were harvested later in the season, 2 months after the others. Once bunya nuts are dispersed on the ground, they can start to degrade, due to the moist environment (Vesoul & Cock, 2012). This may have influenced the earthy, herbal, and chemical aromas and flavors showed in PC1 and the strong intensity of these attributes. The combination of these two factors supports the hypothesis that the differences found in the samples depend mainly on how they are prepared or whether they are chestnuts or bunya nuts, that is, different species.

Bunya nuts collected from different regions as indicated by: Blackbutt (BTT); Landsborough (LBH); Bunya Mountains (BMS); Toowomba (TW). Chestnut samples (CH). Processing types indicated as boiled (B) and roasted (R). Year of collection indicated (2019 ad 2020).

Bunya nuts are hard at first bite, slightly less hard than Brazil nuts and, during mastication, they may feel crumbly and grainy, similarly to macadamias, although dry, possibly because of its high starch content. They have a savory aroma and flavor, similar to cooked potato, as well as a roasted aroma, similar to toasted nuts and popcorn. Bunya nuts may taste and smell slightly sweet and develop sweet linger when swallowed. Some nuts can show a combination of slightly chemical, herbal (a combination of parsley and eucalyptus), and earthy (as root vegetables) aroma and flavors, ending with an earthy aftertaste. They are slightly hard to clear and leave a drying and numbing, almost metallic, sensation after swallowing.

### 3.4 Sensory differences among bunya nut and chestnut samples

**FIGURE 3:**
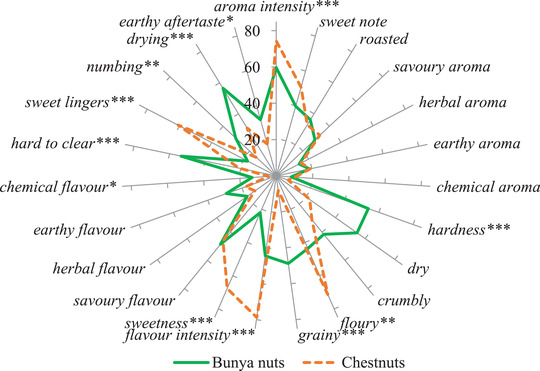
Sensory profiles of bunya nuts and chestnuts (*n* = 4 replicates × 14 panelists, scale of 0–100). Significant *F*‐ratios are indicated by *(*p* < 0.1), **(*p* < 0.01), ***(*p* < 0.001) and ns: not significant (*p* > 0.1)

The cob‐web plot in Figure 3 shows the main differences in sensory properties of bunya nuts and chestnuts.

Both chestnuts and bunya nuts had a sweet note aroma, but chestnuts were much sweeter in flavor (68 vs. 22) and left a strong sweet linger sensation (61 vs. 18) after swallowed. The intensity of flavors and aromas in bunya nuts were not as strong as in chestnuts. In terms of texture, chestnuts were slightly more floury. In contrast, bunya nuts were harder to bite (54 vs. 7), and grainier (49 vs. 8). Bunya nuts were also more difficult to clear from the mouth and teeth and, after swallowing, they left a slightly numbing sensation, differently from chestnuts, where *numbing* was very low or nonexistent.

Regarding the composition of these two types of nuts, the sugar profile showed differences. Even though fructose is usually considered to have a slightly stronger relationship with *sweetness* when compared to sucrose (Portmann et al., 1992), the values for fructose in both nuts were very low and sucrose seems to have a stronger effect on sweetness. The total soluble sugars, especially sucrose, was higher for chestnuts and this could explain why chestnuts showed higher scores for *sweet note*, *sweetness*, and *sweet linger* when compared to bunya nuts. Chestnuts are also higher in moisture for both boiled and roasted nuts, which explains why these nuts felt less dry than bunya nuts during the sensory sessions. When the nuts are boiled or roasted, the starch gelatinizes, and when this thermal treatment is followed by cooling, the starch retrogrades, increasing firmness and rigidity (Perez‐Rea & Antezana‐Gomez, 2018), which means the nut may become slightly harder to bite. This could be related to the bunya nuts being harder and grainy, while chestnuts were more floury.

When panelists were asked to rate *other aroma* and *other flavor* and mention which different aromas and flavors they might have perceived, the rating for chestnuts was higher, probably because the panelists could perceive the differences between bunya nuts and chestnuts. Also, the attribute “*fishy*” appeared 21 times for describing chestnuts during the three sessions.

### 3.5 Effect of preparation method on the sensory profile of bunya nuts

**FIGURE 4:**
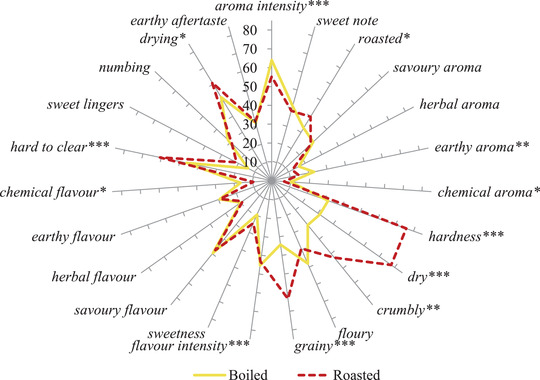
Sensory profiles of boiled and roasted bunya nuts (*n* = 4 replicates × 14 panelists, scale of 0–100). Significant F‐ratios are indicated by *(*p* < 0.1), **(*p* < 0.01), ***(*p* < 0.001) and ns: not significant (*p* > 0.1). Graph lines represent 280 roasted nuts and 280 boiled nuts

The differences in the sensory attributes of the bunya nuts after roasting and boiling is showed in the cob‐web plot in Figure 4.

Differences between roasted and boiled bunya nuts were not as perceptible in terms of aroma and flavor as in texture. The aroma and flavor intensities were slightly higher for boiled samples. This may be because the nuts are placed in the closed plastic cup container right after peeling and the excess water continues to evaporate, releasing some aromas, for example *earthy*, that get trapped inside the cup, even while in the fridge. Roasted samples had a higher score for roasted aroma (40 vs. 33). When the nuts are roasted, Maillard reactions may occur and these reactions are often related to a roasted and sweet aroma, for example chicken and caramel (Wong et al., 2008). Maillard reactions are not only related to aroma and flavor, but also to cross‐linking between proteins or protein and carbohydrates, which contributes to hardness (Starowicz & Zieliński, 2019). This could be related to roasted samples being harder (76 vs. 32) and more difficult to clear from the mouth and teeth. Lastly, the loss and gain of water when roasting and boiling the nuts, respectively, resulted in the roasted nuts being drier than boiled (78 vs. 31) and, consequently, grainier (63 vs. 34) and slightly crumblier.

Collecting the bunya nut samples is a challenge, not only because they are only available once a year in very specific regions, but also that if they are not properly handled and stored, they might have a short shelf‐life due to their high moisture and high water activity, which could make them susceptible to mold growth. In addition, the cones fall in different periods of time, from December to March, making it difficult to calculate time spent on the ground prior to collection. Another challenging aspect of this study was that it was not possible to identify the exact tree from which the nuts originated. Bunya trees grow up to 45 m and the soccer ball sized cones may fall from heights of 30 or 40 m and come to rest some distance from the tree, often exploding and dispersing seeds along the path it takes. Furthermore, commercial bunya nuts, are not from cloned commercial varieties (as with other horticultural produce), but rather would be considered a wild‐harvested product, with inherent variation, even when coming from the same tree, forest, or same region.

## 4 CONCLUSIONS

A lexicon containing 23 attributes was established to describe Australian Araucaria nuts (Bunya nuts) in terms of flavor, aroma, texture, and aftertaste. Overall, bunya nuts were profiled sensorially as intensely *savory* (aroma and flavor), *roasted*, *grainy*, *crumbly*, *hard*, *dry*, *numbing*, *hard to clear*, and *drying*, with subtle *earthy*, *herbal*, and *chemical* flavor and aroma notes, related especially to the late harvest samples from 2020 (Toowoomba origin), which may have been caused by the time of harvest. Bunya nuts are less intense in terms of aroma and flavor than chestnuts, and were significantly less sweet in aroma, flavor, and aftertaste. Texturally bunya nuts were less *floury* than chestnuts but were scored higher for *hardness*, *graininess*, *drying*, and *hard to clear*. The attribute *numbing* mouthfeel and *earthy aftertaste* were also subtle characteristic of bunya nuts that differentiated them from chestnuts. A comparison between roasted and boiled samples suggested that the main differences were related to texture. Roasted samples were scored higher for hardness, *dry* and *drying* mouthfeel, *crumbly*, and *graininess*. Roasted bunya nuts also scored higher for *roasted* (aroma) and *sweet linger* aftertaste.

Bunya nuts can be used as a suitable food source with an interesting flavor and aroma profile as a whole nut. They can be prepared using the two different methods aforementioned. In the future, products developed with bunya nuts can take into consideration the different aspects discussed in this study. Further research may include the comparison to nuts from *A. angustifolia* and *A. araucana* species, and also the study on the sensory properties of bunya nuts after being subjected to different processing techniques, such as fermentation and drying and grinding to produce flour. Bunya nut flour may also present unique sensory characteristics and can be further utilized to produce bakery products and extruded snacks. Chemical analysis of volatile compounds and mechanical measurements of texture could also be studied in the future and related to the sensory results found in this study.

## AUTHOR CONTRIBUTIONS

Jaqueline Moura Nadolny: Data curation; formal analysis; investigation; methodology; validation; visualization; writing – original draft. Odette Best: Investigation; supervision; writing – review & editing. Emma Hassall: Formal analysis; resources; software.

## CONFLICT OF INTEREST

The authors declare no conflict of interest.

## Supporting information

Table S1 Summary of panelist performance, discrimination power and repeatability determined from an ANOVA model of the sensory data obtained for samples. (*n* = 12 samples x 4 replicates x 14 panelists)
Table S2 Descriptive analysis (*n* = 12 samples x 4 replicates x 14 panelists).
