# Supplementary material for: Sensory properties of Australian bunya nuts
Source: J Food Sci. 2022 May 20;87(6):2732–43. doi: 10.1111/1750-3841.16184 (PMC9325081; doi:10.1111/1750-3841.16184)
Supplement: Supplementary file 1 — Table S1 Summary of panelist performance, discrimination power and repeatability determined from an ANOVA model of the sensory data obtained for samples. (n = 12 samples x 4 replicates x 14 panelists) Table S2 Descriptive analysis (n = 12 samples x 4 replicates x 14 panelists). [file JFDS-87-2732-s001.docx]

Supplementary material for the article entitled:

Sensory properties of Australian bunya nuts

Panel performance was assessed including each panellist’s discrimination ability among samples and repeatability across replicates (Table S1).

Table S1 Summary of panellist performance, discrimination power and repeatability determined from an ANOVA model of the sensory data obtained for samples. (n=12 samples x 4 replicates x 14 panellists)

| Panellist | 1 | 2 | 3 | 4 | 5 | 6 | 7 | 8 | 9 | 10 | 11 | 12 | 13 | 14 |
| --- | --- | --- | --- | --- | --- | --- | --- | --- | --- | --- | --- | --- | --- | --- |
| Discrimination Power | 11 | 13 | 14 | 10 | 13 | 13 | 16 | 16 | 15 | 11 | 16 | 13 | 14 | 8 |
| Repeatability | 17 | 17 | 18 | 19 | 19 | 20 | 18 | 19 | 17 | 16 | 18 | 21 | 18 | 19 |
| Total^1^ | 28 | 30 | 32 | 29 | 32 | 33 | 34 | 35 | 32 | 27 | 34 | 34 | 32 | 27 |

^1^The ideal total value is 46.

Attributes *other aroma* and *other flavour* were excluded from panel evaluation analysis.

Across the 12 samples and 4 replicates, 10 of the 14 participants were able to differentiate at least 13 of the 23 attributes. In terms of repeatability, all panellists were able to repeat the value for the same replicates for at least 16 of the attributes.

A summary of the minimum, maximum, mean, standard deviation (SD), coefficient of variation (CV %) and standard error of the mean (SEM) for each of the sensory attributes were calculated (Table S2) to determine panel performance in terms of how the samples were distributed and how the scales were used.

Table S2 Descriptive analysis (n=12 samples x 4 replicates x 14 panellists).

| Attribute | Minimum | Maximum | Mean | SD | CV% | SEM |
| --- | --- | --- | --- | --- | --- | --- |
| Aroma (low – high, 0-100) | | | | | | |
| *aroma intensity* | 48 | 80 | 62 | 11 | 17 | 3.1 |
| *sweet note* | 31 | 51 | 42 | 6 | 15 | 1.8 |
| *roasted* | 22 | 52 | 36 | 10 | 28 | 2.9 |
| *savoury* | 21 | 39 | 30 | 7 | 23 | 1.9 |
| *herbal* | 4 | 29 | 14 | 7 | 51 | 2.1 |
| *earthy* | 5 | 56 | 19 | 14 | 74 | 4.0 |
| *chemical* | 2 | 19 | 9 | 5 | 58 | 1.4 |
| *other aroma* | 0 | 33 | 6 | 10 | 157 | 2.8 |
| In-mouth texture (low – high, 0-100). *Hardness* (soft - hard, 0-100), *dry* (moist - dry, 0-100) | | | | | | |
| *hardness* | 0 | 82 | 46 | 29 | 62 | 8.3 |
| *dry* | 9 | 82 | 49 | 26 | 54 | 7.6 |
| *crumbly* | 23 | 60 | 40 | 13 | 32 | 3.7 |
| *floury* | 34 | 76 | 48 | 12 | 25 | 3.5 |
| *grainy* | 0 | 68 | 42 | 22 | 51 | 6.2 |
| Flavour (low – high, 0-100) | | | | | | |
| *flavour intensity* | 35 | 79 | 50 | 15 | 30 | 4.3 |
| *sweetness* | 7 | 73 | 30 | 19 | 65 | 5.6 |
| *savoury* | 41 | 54 | 48 | 5 | 10 | 1.4 |
| *herbal* | 11 | 35 | 19 | 7 | 38 | 2.0 |
| *earthy* | 18 | 60 | 27 | 13 | 48 | 3.8 |
| *chemical* | 4 | 20 | 12 | 6 | 55 | 1.9 |
| *other flavour* | 0 | 25 | 6 | 9 | 150 | 2.5 |
| Aftertaste and mouth-feel (low – high, 0-100). *Hard to clear* (easy to clear - hard to clear, 0-100) | | | | | | |
| *hard to clear* | 14 | 66 | 48 | 16 | 33 | 4.6 |
| *sweet linger* | 5 | 69 | 25 | 18 | 74 | 5.3 |
| *numbing* | 14 | 37 | 28 | 7 | 24 | 1.9 |
| *drying* | 28 | 65 | 52 | 11 | 22 | 3.3 |
| *earthy* | 18 | 56 | 30 | 12 | 39 | 3.4 |

Overall, the scale was well used for most attributes and variability was observed among samples. Aroma attribute sweet note and flavour attribute savoury had the lowest CV% and small differences between minimum and maximum values, which means they had the lowest contribution to the discrimination.
